# Primary caregivers, healthcare workers, teachers and community leaders’ perceptions and experiences of their involvement, practice and challenges of disclosure of HIV status to children living with HIV in Malawi: a qualitative study

**DOI:** 10.1186/s12889-018-5820-z

**Published:** 2018-07-16

**Authors:** Fatch W. Kalembo, Garth E. Kendall, Mohammed Ali, Angela F. Chimwaza, Mary M. Tallon

**Affiliations:** 1 School of Nursing, Midwifery and Paramedicine, Curtin University, Perth, Australia; 2 Mzuzu University, Mzuzu, Malawi; 3 Kamuzu College of Nursing, University of Malawi, Blantyre, Malawi

**Keywords:** HIV disclosure, Children, Perceptions, experiences, working together

## Abstract

**Background:**

The World Health Organisation has recommended that healthcare workers, teachers and community leaders work with parents to support children living with HIV. The aim of this study was to assess the perceptions and experiences of primary caregivers and other care providers such as healthcare workers, teachers, and community leaders regarding their involvement, practice and challenges of HIV disclosure to children aged between 6 and 12 years living with HIV in Malawi.

**Methods:**

Twelve focus group discussions and 19 one-on-one interviews involving a total of 106 participants were conducted in all three administrative regions of Malawi. The interviews and focus group discussions explored perceptions and experiences regarding involvement, practice and challenges of disclosure of HIV status to children. Data were analysed using thematic analysis.

**Results:**

Primary caregivers, healthcare workers, teachers, and community leaders all reported that the disclosure of HIV status to children was not well coordinated because each of the groups of participants was working in isolation instead of working as a team. A “working together” model emerged from the data analysis where participants expressed the need for them to work as a team in order to promote safe and effective HIV status disclosure through talking about HIV, sharing responsibility and open communication. Participants reported that by working together, the team members would ensure that the prevalence of HIV disclosure to young children increases and that there would be a reduction in any negative impact of disclosure.

**Conclusion:**

Global resources are required to better support children living with HIV and their families. Healthcare workers and teachers would benefit greatly from training in working together with families living with HIV and, specifically, training in the disclosure process. Resources, in the form of books and other educational materials, would help them explain HIV and its effective management to children and families.

## Background

The prevalence of HIV disclosure to children remains very low in sub-Saharan African countries despite the World Health Organisation’s (WHO) recommendation that children living with HIV should be gradually informed about their HIV status from six through to 12 years of age, according to their level of emotional, social and cognitive development [1]. It is beneficial for children living with HIV to know about their HIV status by the time they reach adolescence as it enhances their adherence to antiretroviral (ARV) medication, participation in ongoing medical care, and psychosocial resilience, and lessens the risk of passing their infection to others through sexual contact [2, 3]. The WHO has also recommended that parents seek support from, and share the disclosure process with, teachers, healthcare workers, and community leaders [1]. There is evidence that health outcomes for children are enhanced when their medical and psychosocial care is shared with supportive adults [4].

Parents are widely considered to have the principle role of telling children about their HIV status. Parents are legally responsible for their children and are considered to have their best interests at heart [5]. They are in the best position to support their child, help them accept their condition, adhere to HIV medication, and regain their self-esteem [6]. Parents have the right to decide whether to disclose to their child or not and if they choose to disclose, they have the right to make decisions regarding when, how, where and who is the best person to disclose [7]. In reality, the majority of parents do not disclose to their child because of concerns about the child’s capacity to understand and their emotional readiness to cope with the diagnosis [8, 9]. Further, they have concerns about bringing stigma and discrimination to the family [10, 11], and concerns about a lack of support from healthcare workers [12].

Indeed, it has been suggested that healthcare workers are the most appropriate people to coordinate supportive care for children because HIV is a focus of their practice [4]. Healthcare workers have knowledge about HIV and technical skills that the other groups do not have [1, 13]. Healthcare workers are the first people to learn of the HIV diagnosis, and they have the responsibility of sharing this confidential information with the child’s parents [14]. Healthcare workers can use their communication skills to help parents disclose to their children, and can share their knowledge and understanding of the condition with teachers and community leaders [14]. Continuous interaction between healthcare workers and children living with HIV has been shown to facilitate children’s acceptance of the condition, as well as improve their resilience [13, 15]. While the participation of healthcare workers in the disclosure process is essential, the authors of recent sub-Sahara African studies have revealed that many are reluctant to do so because of the unwillingness of parents to disclose [12, 16], a lack of training [11, 17, 18], inadequate knowledge and skills, and a lack of disclosure materials [17, 18].

In addition to healthcare workers, school teachers play a role in helping children to adapt to HIV and achieve good academic outcomes [19, 20]. Research has shown that making schools HIV friendly is one of the best ways to provide children living with HIV a safe, protective, caring, and supportive environment [21]. According to the United Nations Educational, Scientific and Cultural Organisation (UNESCO), schools should provide education, counselling, and psychosocial support, and assist children to access adequate nutrition and healthcare services [20]. This is crucial because children living with HIV face a number of challenges at school, including stigma and discrimination from other students, absenteeism due to sickness, a lack of the privacy required for taking HIV medications, and difficulty obtaining permission to attend hospital appointments when teachers are not aware of their HIV status [20, 22–25]. Ideally, school teachers do need to know when children have HIV because the HIV infection can affect motor and neurocognitive development, thereby impacting on academic performance [15]. While schools are supposed to be safe, protective, and caring environments, this is not always the case according to the authors of research conducted in Malawi, Kenya, and Zimbabwe [22, 26].

Finally, the communities where children and their families live have been identified as an important source of psychosocial support for children living with HIV in sub-Saharan Africa [10, 27]. Religious leaders, traditional village headmen, the leaders of community-based organisations, and the leaders of support groups can all play an important role in mobilising communities to support children living with HIV [28]. Community leaders can assist in raising awareness of HIV, disseminating information, providing pastoral support to children and their families living with HIV, and promoting the sustainability of their care [29]. In addition, community leaders can advocate for the rights of people living with HIV and help to fight stigma and discrimination [30]. Nonetheless, despite the significant role community leaders are able to play in mobilising community support, they are rarely involved to any great extent [31].

It is estimated that there were 84,000 children (1.6% of the population) under the age of 14 living with HIV in Malawi in 2015 [32]. Of these, 60% were taking ARV medications [32]. While WHO recommends that all children living with HIV should be told of their HIV status, the current prevalence of disclosure to children in Malawi is unknown. There are reports that children in Malawi living with HIV face many challenges including: high levels of poverty, loss of one or both parents, bullying, and stigma and discrimination [33–35]. While it is well known in Malawi that parents, healthcare workers, teachers, and community leaders all play major roles in caring for, and supporting, children living with HIV, no previous research has focused on all these four groups regarding their perceptions and experiences of the disclosure process. This study sought to engage primary caregivers, healthcare workers, teachers, and community leaders in order to assess their perceptions and experiences regarding their involvement, practice and challenges of HIV disclosure to children aged 6–12 years living with HIV in Malawi.

## Methods

### Setting and study participants

Data were collected through one-on-one interviews and focus group discussions conducted with primary caregivers, healthcare workers, teachers, and community leaders recruited from eight districts in the three administrative regions of Malawi; three districts from the South (Nsanje, Mulanje, and Mangochi), three from the Centre (Dowa, Salima, and Kasungu) and two from the North (Mzimba and Karonga). Data were collected from March to June 2015. The eligibility criteria of the participants are presented in Table 1.

**Table 1:** Recruitment criteria and summary of data collection plan

Participants	Sample size	Eligibility criteria	Recruitment location
One-on-one interviews and focus group discussions			
Primary caregivers,	6 focus groups	• Parent of a child living with HIV or someone providing care to a child living with HIV between the ages of 6 to 12 years for more than six months • 18 years or older • Ability to provide informed consent	Antiretroviral therapy clinics
Healthcare workers	7 one-on-one interviews	• Working in children’s antiretroviral therapy clinics • Being a nurse, counsellor or clinician • Ability to provide informed consent	Antiretroviral therapy clinics
Primary school teachers	6 focus groups	• Teaching at a primary school • Ability to provide informed consent	Primary schools surrounding participating hospitals
Community leaders	7 one-on-one interviews	• Living near the participating hospitals • Being 18 years or older • Having a certain responsibility within the community such as being a community-based organisation leader or a village headman • Ability to provide informed consent	Communities surrounding participating hospitals
Adolescents living with HIV	5 one-on-one interviews	• Between 13 to 18 years old • Living with HIV • Aware that they have HIV • Leader of children HIV support groups • Ability to provide informed consent	Antiretroviral therapy clinics Community support groups surrounding participating hospitals

### Procedure

Primary caregivers and teachers participated in focus group discussions while healthcare workers and community leaders participated in one-on-one interviews. The lead researcher facilitated all focus groups and interviews, and a research assistant audio recorded the proceedings. Following informed consent, an interview or focus group discussion guide was used to ensure themes about HIV disclosure were standardised across all participants and groups. Interviews took approximately 30 to 50 min, and focus groups took approximately 45 to 60 min to be completed. The number of interviews and focus group discussions was determined by saturation of data, which was considered to have been reached if there was no new information arising from the interviews or focus groups. Ethics approvals were obtained from the Curtin University Human Research Ethics Committee and the Malawi Government Health Science Research Committee prior to the commencement of data collection. Details about the focus groups and interviews are presented in the following sections.

#### Focus group discussions - primary caregivers and teachers

An arrangement was made with ART clinic staff to inform all primary caregivers of children aged 6 to 12 years who came regularly to the clinic for medication about the research study and requesting their participation. They were approached by a research assistant who asked them if they would like to participate in a focus group discussion about HIV disclosure. The focus group discussions took place in a quiet, private room after the primary caregivers and their children had been seen by the clinic staff. Children were kept separate from the primary caregivers during the focus groups to avoid inadvertent disclosure. They were entertained with cartoon shows on a portable DVD player in a separate room. One of the research assistants was assigned to look after the children for this purpose.

Teachers were recruited from primary schools where student ages ranged between 6 and 12 years. The lead researcher met with the head teacher of each school and following their approval, teachers were informed about the research study. Of approximately 30 teachers in each school, most were willing to participate. The first 6 to 9 individuals, considered to be the optimal number for a focus group [36], who were approached by the researcher were asked to join the focus group. Reflecting the gender distribution of primary school teachers in Malawi, the participants were predominantly female. The focus groups were organised to take place at a convenient time, in a quiet private room at the school.

#### One-on-one interviews - healthcare workers and community leaders

Following approval from the district head officer of each hospital, nurses, clinicians and counsellors who worked in ART clinics were approached to participate in the study. In Malawi, it is usual for each ART clinic to have at least two staff members, one a registered nurse or nurse technician, and a physician or counsellor. Almost all healthcare workers who were approached agreed to participate in the study. Interviews were conducted after working hours.

The group described as community leaders comprised of leaders of HIV community-based organisations and support groups, adolescents living with HIV, and village headmen. Community leaders were recruited from across Malawi with assistance from the District ART clinic staff, the District National Organisation of People Living with HIV and AIDS in Malawi (NAPHAM), and the National Family Planning Association of Malawi (FPAM). FPAM is an organisation that works with youth who are living with HIV while NAPHAM is a support organisation for all people living with HIV in Malawi. The management staff of the District ART Clinic, FPAM and NAPHAM provided names and contact information for the community leaders who were subsequently contacted, briefed about the study objectives, and invited to participate. Dates and venues for the interviews were arranged for those who expressed interest in participating. There were no refusals. Interviews were conducted either at the participant’s home or at one of the offices of the three organisations.

### Interview and focus group guides

Four interview and focus group guides (one each for healthcare workers, primary caregivers, primary school teachers and community leaders) were developed by the research team through the review of literature guided by the study aims and objectives. After initial development, the guides were translated to Chichewa, the local language of Malawi, and then back translated to English by professional translators following the WHO instrument translation process [37]. Once all language issues were corrected the interview and focus group guides were reviewed for readability and interpretation prior to data collection with participants who were excluded from the study sample.

The interview and focus group guides contained questions to elicit the views of participants regarding the disclosure of HIV status to children. The focus group guide for primary caregivers included the following questions: *What are your thoughts about telling children that they have HIV; In your family, who would make a decision to inform your child about his or her HIV status; How should children be informed of their HIV status; What kind of support would you need to help you inform your child of his or her HIV status; What do you think are some of the reasons that prevent primary caregivers from disclosing HIV status to their children;* and *what kind of support would you need to tell your child that she or he has HIV.*

The perspectives and experiences of teachers were explored using a focus group guide which contained the following questions: *From your experience, what are some of the problems faced by children living with HIV at school; What kind of support do you provide to pupils living with HIV; What are your thoughts regarding telling children that they have HIV; What are your thoughts regarding the involvement of teachers in informing children about their HIV status;* and *how can teachers be prepared for the role of telling children that they have HIV.*

The views and experiences of healthcare providers were examined using the following questions as a guide: *What does disclosure of HIV status mean to you; What are your thoughts on how children living with HIV should be told about their condition; What are your thoughts regarding involvement of healthcare workers in disclosure of HIV status to children; What kind of support should be given to a child after being told that she or he has HIV; From your experience what do you think are some of the challenges to disclosure of HIV status to children;* and *from your experience what do you think are some of the facilitators of disclosure of HIV status to children?*

The following questions were asked to explore the perspectives and experiences of participating community leaders: *What do you think are some of the problems faced by children living with HIV in the community; What are your thoughts about telling children that they have HIV; What are your thoughts regarding involvement of community leaders in telling children that they have HIV;* and *How can community leaders promote disclosure of HIV status to children.* Apart from the above questions, adolescents living with HIV were also asked the following questions*: How did you know about your HIV status; How did you react when you were first told that you had HIV; What kind of support did you receive to cope with your HIV status after being told about your HIV status;* and *how should HIV status to children be conducted.*

### Data analysis

The focus group and interview transcripts were analysed using the six steps of thematic analysis described by Braun and Clarke [38]: a) familiarisation with the data, b) coding, c) searching for themes, d) reviewing themes, e) defining and naming themes, and f) writing-up.

Audio recordings of the interviews were transcribed by the lead researcher and three research assistants. The lead researcher was involved in the transcription as a way to familiarise himself with the data. Since the recordings were in Chichewa, the Malawian local language, data from the recordings were first transcribed into Chichewa prior to translation into English. Before translation of the Chichewa data, the lead researcher who is fluent in the language verified the transcription by re-reading the transcribed data while listening to the recorded data. Throughout data collection, the lead researcher recorded reflexive notes to bracket out any preconceived ideas and examine the data in its authentic form. Field notes were also recorded during the interview and focus groups. These notes contained objective descriptions about the context that were not captured in the transcripts. Both reflexive and field notes were referred to throughout the analysis process. Participants were de-identified and pseudonyms chosen by the research team.

Through the process of reading and re-reading the transcripts, the lead researcher identified and developed a list of codes which were organised using a table. An independent researcher with experience in qualitative research, but who had no knowledge of the research, was asked to review the transcripts and the identified codes to confirm if the codes were arising from the transcripts and to verify that all important codes were captured. To follow, the identified codes were examined and re-examined by the research team during regular meetings with the lead researcher. Some codes were added while others were removed depending on the interpretations of the collective team.

The codes were then examined for commonalities and grouped accordingly. A table was used to organise and represent the codes into groups. During this process of interpretation, some of the grouped codes were combined, some were changed, and others were discarded. These groups were examined and re-examined by the lead researcher and the independent researcher who initially reviewed the codes and emerging themes were identified. These emergent themes were discussed with the collective research team during regular meetings. Some were found to be principal and overarching emergent themes that were integrally linked to others, while other themes were found to represent prominent aspects within a bigger emergent theme. These emergent themes were named and identified as main themes or subthemes, and a conceptual model was constructed.

The named themes and sub-themes from the data were found to resemble the reciprocal interaction between the child, family, and community, and the physical contexts of the child’s environment as represented in Bronfenbrenner and Ceci’s bioecological model [39]. For this reason and given that one of the major limitations of thematic analysis is the inability to describe data in detail if a theoretical framework is not used [38], this bio-ecological model of early child development was used to inform the construction of the final emergent conceptual model.

A detailed account of the findings from the analysis was then written up in a report that was guided by the emergent conceptual model and supported by related quotations and detailed description of the data.

## Results

In all, 12 focus group discussions and 19 interviews were conducted involving a total of 106 participants. The demographic characteristics of the study participants are presented in Table 2. Of the 106, 42 were primary caregivers, seven healthcare workers, 45 teachers, seven community leaders, and five were adolescents living with HIV who were also support group leaders. The mean ages of primary caregivers, healthcare workers, teachers, community leaders and adolescents living with HIV were 44, 41, 37, 46 and 15 years respectively. In terms of gender, females were the majority across all groups of participants except the community leaders where males predominated.

**Table 2:** Demographic characteristics of study participants (N = 106)

Characteristic	n (%)
Primary caregivers	*n* = 42
Primary caregiver’s Age	
Age range in years (Mean)	18–69 (M = 44)
Age of the primary caregiver’s child	
Age range in years (Mean)	6–12 (M = 10)
Sex	
Male	8 (19)
Female	34 (81)
Relationship to the child	
Biological mother	24 (57)
Biological father	7 (17)
Grandparent	8 (19)
Others	3 (7)
Education level	
No education	12 (29)
Primary	21 (50)
Secondary	5 (12)
College/ university	4 (9)
Occupational status	
No employment	8 (19)
Farming	19 (45)
Self-employment	9 (22)
Employment	6 (14)
Healthcare workers	*n* = 7
Age	
Age range in years (Mean)	32–52 (41)
Male	2 (29)
Female	5 (71)
Professional group	
Registered nurse	2 (29)
Nurse technician	3 (43)
Counsellors	1 (14)
Clinician	1 (14)
Working experience in ART clinic	
Range in years (Mean)	1–7 (M = 3)
Characteristic	n (%)
Teachers	*n* = 45
Age	
Age range in years (Mean)	31–48 (M = 37)
Sex	
Male	12 (27)
Female	33 (73)
Level of grade teaching	
Grade 1–3	16 (36)
Grade 4–6	19 (42)
Grade 7–8	10 (22)
Teaching experience	
Range in years (Mean)	3–16 (M = 7)
Community leaders	n = 7
Age	
Age range in years (Mean)	41–53 (M = 46)
Sex	
Male	5 (71)
Female	2 (29)
Type of community leader	
Community based organisation	5 (71)
Traditional leaders	2 (29)
Adolescents living with HIV	*n* = 5
Age	
Age range in years (Mean)	13–18 (M = 15)
Sex	
Male	1 (20)
Female	4 (80)
Education level	
No education	1 (20)
Primary	2 (40)
Secondary	2 (40)
Duration since HIV disclosure	
Range in years (Mean)	1–3 (M = 2)

**Fig. 1:**
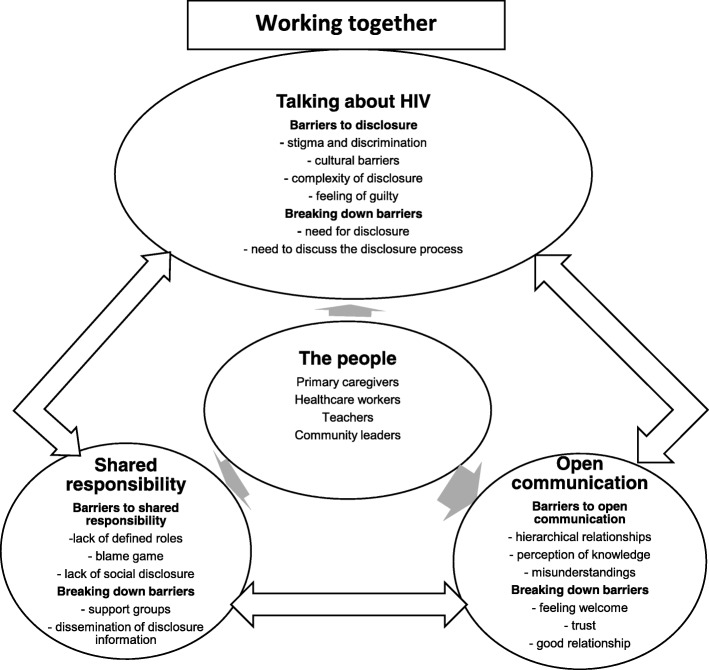
The Working together model

Initially, data for the four groups of participants (primary caregivers, healthcare workers teachers and community leaders) were analysed separately. The themes that emerged from transcripts across all four groups were found to be very similar. Therefore, data were combined for further analysis while extracts identified group membership. Three themes were identified from the analysis. These themes were named: ‘talking about HIV’, ‘shared responsibility’, and ‘open communication’. Together, these three themes characterised a conceptual model that was named ‘working together’ (see Fig. 1).

### “Working together” conceptual model

A conceptual model was constructed based on the data emerging from the thematic analysis of transcripts where participants emphasised the need for all group of participants to work together in order to promote the practice of effective HIV status disclosure to children. There was a great deal of discussion related to working together among the study participants. Primary caregivers, healthcare workers, teachers, and community leaders all felt that the disclosure of HIV status to children was not well coordinated because each of the stakeholders was working in isolation rather than working as a team. All participants expressed the need to work together when talking about HIV and disclosure. This was found to include sharing the responsibility to ensure that the process of HIV disclosure was conducted in a coordinated way and ensuring that there was open communication as they carried out their respective tasks (see Fig. 1). These three themes are described in greater detail below. Pseudonyms chosen by the research team are used to present direct quotes from participants.

### Theme one: Talking about HIV

Several primary caregivers and teachers said that they welcomed the opportunity to meet together to talk about HIV and disclosure to children. Aida, the mother of one child, said that: *“This forum has benefited me a lot and I will start getting free with him and start disclosing slowly.”* Patuma, another mother said that: “*Today, I had the opportunity to learn about what to tell her based on what my friends are saying here. I think I have found an answer.”* One of the teachers, Yosefe, made the following comment:

“I am ... happy that ... you have involved us at the grassroots [level] because ... most of you like to just involve healthcare workers and leave us out in programmes affecting children, and you find that such programmes are not effective because you missed important issues that could have been included if you had involved us.”

Participants reported several reasons why HIV disclosure was not often discussed. These reasons and participants’ suggestions for breaking down barriers are presented in the following subthemes.

#### Reasons for not disclosing HIV

While most participants recognised the importance of HIV disclosure to the child, they reported that disclosure was rarely practised because of the fear of stigma and discrimination, cultural sensitivity to sexual topics, the complexity of the disclosure process, and primary caregiver’s feelings of guilt about transmitting the virus to the child.

##### Stigma and discrimination

Stigma and discrimination against people living with HIV were reported by most of the participants as the main reason for non-disclosure. Sainabu, a mother of a seven year old child said that “*We are afraid to tell a young child of seven years about his HIV status because he may end up telling his friends about his condition who may then discriminate against him.*” Joyce, a mother of a ten year old, explained how her child was about to drop out of school because of stigma. One nurse reported that, in her experience, stigma and discrimination against people who were known to be HIV positive were still common in some communities. Mrs. Banda, a teacher, reported how children suspected of having HIV were stigmatised at school *“When we are teaching, you find that some of the children start mentioning names of the pupils they suspect to have HIV, they are like…madam, this one has HIV.*” In addition, Yohane, an adolescent living with HIV explained how the experience of stigma and discrimination in her community affected her:

“*The problem is that when we are going to the hospital to collect medication, people near our home call us names like …. look at HIV positive children, they are going to the hospital to receive ARVs…. We are always sad with this.”*

Another adolescent, Sainabu, reported that he had not told anyone about his HIV status because of fear of stigma and discrimination. *“I have not told anybody about my HIV condition because I am afraid that they can start discriminating against me”.*

##### Cultural sensitivity to sexual topics

Many participants thought that cultural practices that discouraged parents from discussing HIV sexual related topics were barriers to HIV status disclosure to children. One community based leader reported that “*it is difficult for parents to disclose HIV to a child because of culture…when parents are telling a child about HIV, a child can be surprised and say what are my parents trying to tell me?”* Many healthcare workers also reported that some parents felt uncomfortable discussing the issue of HIV with their young child because it was related to sex. “*Some parents find it difficult to discuss reproductive health issues with their children despite being important for children with this condition.”* Tamala, a mother of a 12 year old child, reported that the mode of HIV transmission made it hard for a primary caregiver to discuss the disease with the child “*This is a difficult issue to discuss with the child because of the way the child got the infection.”*

##### Complexity of disclosure

Many healthcare workers, teachers and community leaders acknowledged that explaining HIV to the child was a difficult task which required confidence, and skills to initiate trust. Lesinati, a teacher, felt that disclosure was a difficult task because of the poor outcomes of HIV infection. “*If you have been diagnosed with HIV, it marks the end of your life, so you have to take care of how you inform the child about his condition. It requires you to go along very well with the child, it is, of course, a long process.”* One of the nurses said that some primary caregivers do not know how to disclose to the child. *“it is a difficult issue … they have problems to inform their child about her/his HIV status.”*

##### Guilt and fear of family disharmony

Most healthcare workers and teachers thought that many of the children who had HIV acquired the infection from their mother and that many primary caregivers felt guilty and did not want to disclose to their child for fear of bringing disharmony to their homes. Patuma, a mother of a 10 year old boy said that:

“Sometimes when you tell your child about his/her HIV status, for instance, a 12-year-old girl, she may ask you….I have never slept with a man how did I get this disease?.…. so this causes conflicts.”

One of the adolescents, Shaibu, made the following comment:

“It is a different case if you got the infection because you were involved in promiscuous behaviour, you can blame yourself, but getting it from parents as I did is difficult to understand. I had no chance to confront my parents about this because I lost them when I was young”*.*

#### Breaking down barriers

Most of the participants felt that it was important to break down the barriers to talking about HIV disclosure. They acknowledged the need to disclose HIV status to a child and to discuss the disclosure process.

##### The need for disclosure

Many participants reported that it was necessary to inform children of their HIV status because doing so would help to protect children from reinfection, promote autonomy in care and treatment as well as help children to live a healthy life. The majority of participants felt that disclosure would help children to understand about the disease and treatment. Mr. Sauli, the father of an eight old child, said that “*It is important to explain to the child while he is still young so that he can grow up knowing his disease and the medications that he is taking.”* One of the counsellors reported that “*when a child is aware of his HIV status, he adheres to ARVs prescription… because he knows the benefits of the medication.”* Mrs. Khoma, a teacher, said that disclosure can protect the child and others from HIV infection*. “If we tell the child while young, he cannot spread the infection to others and he can also know how to protect others from the infection****.”*** On the other hand, the majority of the adolescents interviewed thought that children should be told about their HIV status starting from the age of six years so that they can grow up knowing their HIV status. *“The child should be told about his or her condition from the age of six years going up yeah…. because by then they can talk and even understand issues.” (Basamu, adolescent living with HIV).*

##### Need to discuss the disclosure process

Participants had different views regarding the best age and person to disclose as well as how the disclosure process should be conducted. The majority of participants identified primary caregivers as the principle people to disclose HIV to the child with support from healthcare workers and others. One of the nurses said that *“The guardian or parent of the child should be the one to disclose to the child because she is the one who stays with the child, they are close but also they trust each other.”* Primary caregivers went further to suggest the person in the family closest to the child should disclose. Some participants thought that primary caregivers and healthcare workers should disclose to the child together. Chifundo, a mother of a 6 year old child said that *“The best way would be for a parent and healthcare worker to come together and inform the child about his/her HIV status. In this way, the child can easily understand his/her condition.”* On the other hand, some thought that any trustworthy or responsible person could disclose to the child. Mr. Jere, a primary school teacher, suggested that “*Sometimes as a parent, you can be uncomfortable to explain this to a child, and you can ask people that you trust to do that for you.”* In addition, there were some participants who were of the opinion that community leaders, primary caregivers, teachers and healthcare workers should all take part in the disclosure process. Janet, a nurse, commented that *“Anyone who is responsible and who has knowledge can disclose to the child be it parents, community leaders or teachers.”*

### Theme two: Open communication

The majority of the participants expressed concern about a lack of openness among the stakeholders to discuss the child’s HIV status and issues regarding disclosure of HIV status. They characterised their communication as closed as opposed to open. Differences of opinions were identified among the participants during discussion about how HIV disclosure to a child should be conducted. Most healthcare workers, teachers and community leaders reported that primary caregivers were not open to discussing issues related to disclosure. This was reinforced by Madalitso, a teacher: “*Most parents are not comfortable to discuss this issue with us.”* More than half of the healthcare workers reported that many primary caregivers were opposed to disclosure of HIV to a child and that they were not open to give reasons for their decision. All adolescents reported that their primary caregivers knew they were living with HIV but were not open to disclose this to them. This was illustrated by one adolescent, Yohane,who stated: *“I was told about my HIV status when I was 12 years old. My parents did not want to tell me about my HIV status despite asking them to tell me the reason for my frequent hospital visits…... one day, I went to the hospital alone and asked the doctor about my condition. The doctor did not hide anything from me; he told me that I had HIV.”*

On the other hand, some primary caregivers reported that healthcare workers did not provide time to discuss issues regarding HIV disclosure to their children. Instead, they were just told to administer medications to the child. Chifundo commented that “*The healthcare workers just tell us about how the child should be taking the medication but do not have time to discuss with us how we are supposed to tell the children about their HIV status*.” A number of primary caregivers reported that they wished they had discussions on safe HIV disclosure with healthcare workers.

#### Barriers to open communication

While many participants expressed their wishes to have more open communications with the other groups of participants, they pointed out that the hierarchical relationships between them and a perceived lack of knowledge and understanding about HIV among the primary caregivers were major constraints.

##### Hierarchical relationships

Although no participant spoke directly about hierarchical relationships, this tension was clearly identified from their comments. Most healthcare workers, teachers, and community leaders expressed a sense of superiority that their knowledge about HIV and health surpassed that of primary caregivers who mostly lacked health education and knowledge especially in relation to HIV. Mr. Salijeni, a grade four teacher, said that *“Teachers have more HIV knowledge than parents. If they can be involved in the disclosure process, they can assist the child to understand about his/her condition.”* Indeed, some healthcare workers were of the view that primary caregivers should follow what they were told to do. Ephraim, a counsellor, reported that “*Parents lack necessary knowledge and skills to disclose, as such, they are supposed to closely follow what we tell them regarding disclosure so that they can assist the child to understand his/her condition.”* Another healthcare worker reported that they instruct primary caregivers to initiate the disclosure process first before they are fully involved in the process. Janet, a nurse, commented that “*When we find that the child is not yet told about his HIV status, we tell the parents to disclose and then hand over the child to us for counselling and teaching about HIV medication.”* Reinforcing this hierarchical relationship, most primary caregivers indicated that they complied with the instructions of healthcare workers, acknowledging the latter’s expertise. Sayinatu said that “*For us to give proper support to a child with HIV, we need to follow what the doctors say.”*

##### Perceptions of knowledge

There was general agreement among all participants that healthcare workers were most knowledgeable about HIV, followed by teachers, community leaders, and then primary caregivers. As such, there was a view that primary caregivers were supposed to listen and do what they were told by healthcare workers, teachers, and others. Thumbiko, a teacher reported that “*Parents do not know the approach that they can use to tell the child that he is HIV positive and that is why healthcare workers should teach them how to disclose*.” This was also supported by a nurse: “*Some parents are illiterate … they lack expertise on how best to disclose to a child.”*

##### Misunderstandings

A hierarchical approach as also evident in the reports by participants that there were often misunderstandings among them regarding disclosure of HIV status to the child. Healthcare workers reported that it was difficult to comprehend that despite repeating the need to disclose to the child, parents were reluctant to do so. “*We keep on reminding them to disclose to the child, but they do not see the need for the child to know, so we give them time to decide when they are ready to disclose.*” On the other hand, many primary caregivers reported that healthcare workers did not seem to understand why they had problems disclosing to their child. Meliya, the mother of a 12 year old child explained: “*Healthcare workers always ask us why we have not disclosed, but they do not understand what it is like to have a child with HIV and to have your neighbours talk about yo*u.” Then again, many teachers stated that they did not understand why primary caregivers were not open to discuss the child’s HIV status with their teacher despite being told by their healthcare workers to do so.

#### Breaking down barriers

Throughout the interviews and focus group discussions, many participants expressed their wish for open communication among all those involved, thereby creating an environment where everyone would feel welcome, and trust could be built, and relationships improved. Siyambota, a community leader, summed up the need for open communication among the people involved in the care of a child: *“I think that if healthcare workers and all the stakeholders can meet and discuss how best this issue can be tackled, then I am sure we can have a good plan on how to assist children in knowing their HIV status.”*

##### Feeling welcome

Many healthcare workers reported that some primary caregivers and their children were avoiding hospitals near their home where they might be known and going to distant hospitals where they were less likely to be judged and felt welcome to receive medication. A few primary caregivers reported that they knew of friends whose children had HIV who were not taking their child to the hospital because they believed they would not be welcomed there. “*Some parents avoid public hospitals because they are not sure how they will be treated, but we still encourage them to go to the hospital to get help.”*

##### Trust

Trust was proposed by some participants as an essential component of a relationship that would ensure open communication among the members. Some healthcare workers thought that primary caregivers did not trust them to support their children. Janet, a nurse, commented: *“Some primary caregivers are not comfortable to let healthcare workers disclose HIV status to their children because they do not know how the disclosure process would be conducted”*. One teacher, Sikawa, stated that all stakeholders needed to trust each other if they were to provide an effective HIV disclosure *“For the whole process of HIV disclosure to be possible, then parents, healthcare workers, teachers and leaders need to trust each other*.” Chiona, one of the traditional leaders, also alluded to this by saying*: “Parents need to trust us chiefs by informing us of the problems their children are facing in the community. Otherwise, it is difficult for us to know.”*

##### Good relationships

All the participants expressed the importance of a good relationship if they were to freely discuss issues related to the child’s HIV status. Some primary caregivers reported that it was important to have a good relationship with teachers and healthcare workers so that they can help to take care of their children*.* Jasimini said that *“We need to get along very well with teachers and healthcare workers because they help in caring for our children.”* A number of healthcare workers said that a good relationship with primary caregivers could facilitate disclosure of HIV to the child. A counsellor, Mr. Nyirenda, commented that “*Effective disclosure depends on the relationship between the healthcare worker and the primary caregiver if there is a good relationship between these two, discussion on HIV disclosure is not difficult.”* Some primary caregivers reported that they had an open discussion with their child’s teacher regarding ways the teacher could assist their child and this was helpful. Eluby, the mother of a 12 year old child explained that:

“*When my child started getting HIV medication, some of her friends were bullying her at school; I had a friend who was teaching at the same school, who was also HIV positive. I discussed this with her, and she helped my child by talking to the other children to stop their bullying behaviour.”*

### Theme three: Shared responsibility

Participants recognised that children who are living with HIV needed care and services provided by many groups of people, including their parents, healthcare workers, teachers, traditional leaders, non-government organisation officers, and government officials. The participants felt that through shared responsibility, children living with HIV can be assisted to achieve good health and developmental outcomes. While most participants expressed the wish that there was shared responsibility, they thought that each group was working in isolation and there was little attempt to share responsibility for care.

#### Barriers to sharing responsibility

##### Defined roles

Most participants expressed the belief that each stakeholder had a specific role to play in the care of a child. They were of the view that primary caregivers had the main responsibility of caring for the child. Esime explained that “*I as a parent, have that main responsibility of providing care that a child needs every day because I am the one who has raised him.”* This was supported by one of the nurses, Janet, who commented that “*the parent has the main responsibility of providing care to a child including giving the child HIV medications.*” The majority of participants were also of the view that healthcare workers had the responsibility of assisting and providing support to primary caregivers to effectively care for their child. With regard to teachers, many participants felt that teachers had a responsibility to protect the child from stigma at school, support the child to attend hospital appointments, and provide additional teaching support. Mr. Mbewe, a teacher, commented: *“We also guide these children and discourage other children who have no HIV from stigmatising or discriminating against these children.*” Some participants thought that community leaders had the responsibility of providing education for people in the community about disclosure and encouraging parents to send their children to support groups. This was illustrated by Adna, a support group leader:

“As a support group we can ensure that once children are disclosed to, they are not stigmatized or discriminated against by organising campaigns in the villages…..we can invite people living with HIV and those without to educate them on negative effects of stigma and discrimination. This can change people’s attitudes and behaviour towards children living with HIV.”

##### Blame game

The lack of shared responsibility identified tensions in the data revealing a ‘blame game’ shared among healthcare workers, teachers, primary caregivers and community leaders. Each group blamed another group for failing to take responsibility for caring for children living with HIV. Martha, a nurse commented that primary caregivers do not disclose to their child after being given an explanation as to why they should do so: *“Many children are not aware of the reason they are taking ART medications, we do explain to parents that they should disclose the condition to the child, but most of them say they have not yet done that*.” Many primary caregivers felt that healthcare workers were not helping them to effectively disclose to the child. This was supported by one of the teachers, Mr. Sande, who said, “*The healthcare workers should also take the responsibility of disclosing the condition to the child, they should not just tell parents that this is a secret.”* Teachers and community leaders reported that although they wanted to take part in the disclosure process, their hands were tied because it was difficult for them to identify children who were HIV positive in order to take part in the disclosure process as most often primary caregivers were not telling them about their child’s HIV status for fear of discrimination. Wanangwa, a teacher’ said that *“Parents are not free to give information to the school about their children who have HIV, which makes it difficult for us, teachers, to identify these children and get involved in the disclosure process.”*

#### Breaking down barriers

Most participants pointed out that shared responsibility had many benefits and there was a need to identify factors that can promote shared responsibility among all groups*.* The majority of primary caregivers felt that shared responsibility would help to ensure continuity of care and appropriate disclosure of HIV status was provided to children. Rhoda, a teacher, went on to say:

“What matters to us most is that we want primary caregivers to be free with us, to tell us if their children are HIV positive, in that way we teachers and parents will work together to assist the children and even at school when other pupils want to call them names, they will be afraid of us because we will put in place measures to protect such children.”

##### Support groups

A number of participants suggested that the formation of support groups for children living with HIV was a good way of facilitating shared responsibility. Fabiano, a clinician, said that *“We have a Teen Club here at the hospital that helps children with HIV to know more about their health. Children from the age of 12 or 13 can join the club.”* Semati, the parent of a 14 year old child reported, “*It is important that the children should be going to a support group so that they are encouraged and not to be worried that they are the only ones with HIV.”* Some community leaders reported that although the support groups were beneficial to children living with HIV, some primary caregivers were not sending their children to these groups. Mercy, a community based leader, commented:

“There is a need for sensitisation because there are many people including children who tested positive for HIV in the villages but have not yet joined the support groups. They do not understand what it means to have HIV in their body. Many of them just stay at home… they do not want to come to the groups and interact with their friends.”

Most of the adolescents living with HIV who took part in the study reported that they felt extremely anxious when they were told they had HIV, and that their attendance at a support group had helped them to accept their HIV status and adopt healthy behaviours. Alinafe, a 15 year old adolescent living with HIV shared: *“When I joined the support group at FPAM, I met children of my age and my fears disappeared because I thought, why should I be worried when my fellow friends are also living with the same condition…. I realised that having HIV is not the end of one’s life.”*

##### Disseminating information about disclosure,

Many participants expressed the view that there was a need for them to share responsibility for disseminating information about HIV disclosure. Some suggestions for dissemination were: holding campaigns in the communities, using the radio and other media, talking about HIV at church gatherings, and putting up posters in hospital departments. One of the counsellors, Sibakwe, suggested:

“Our tradition demands that we respect the chiefs, so I think the best way is to sensitise the chiefs on this issue, informing them on how stigma and discrimination are negatively impacting on the disclosure of HIV status to children, and asking them for permission to educate people to stop stigma and discrimination against people living with HIV.”

Mr. Phiri, the leader of a community based organisation, added that:

“*We have a drama group which can be used to sensitise people in the community about the importance of HIV disclosure to children.”*

## Discussion

The study findings show that participants expressed the need to work together through talking about HIV, open communication and shared responsibility. Participants reported that they wanted to talk together about HIV disclosure. However, the negative consequences of HIV disclosure, cultural barriers, and complexity of the process often prevented them from doing so. Many participants recognised the importance of HIV disclosure as a way of promoting discussion about HIV. It was clear that much of the communication between them was closed because of hierarchical relationships, misunderstandings, and the perception that parents lacked education and knowledge. Participants also recognised the need for shared responsibility in providing care to a child living with HIV. Each group of participants identified specific roles that they should play in the partnership. Nonetheless, participants blamed one another for failure to take responsibility for carrying out their role.

Participants reported that they wanted to talk about HIV disclosure more, but they were hindered by stigma and discrimination, feelings of guilt for transmitting the virus to the child, cultural factors, and the complexity of the disclosure process. HIV disclosure is a sensitive issue to talk about in Malawi because of the stigma and discrimination directed towards people living with HIV [34, 35, 40]. People living with HIV are discriminated against because many people believe that those with HIV: have been involved in socially unacceptable practices, such as sex work; are not moral; are infectious; and are incurable [40]. Furthermore, it has been found that stigma and discrimination have been directed at all members of families that are affected by HIV, including the children [34, 40]. Similar findings have also been reported in other sub-Saharan African countries [9, 11, 41]. In addition, it is difficult for primary caregivers to discuss HIV disclosure with their child because doing so involves talking about sex which is considered culturally inappropriate in Malawi [34].

The lack of confidence about discussing disclosure with their children that was evident among most primary caregivers in this study is another major barrier to HIV disclosure that has been reported in previous studies [12, 42–44]. These findings highlight the crucial need for healthcare workers to support primary caregivers appropriately through the disclosure process. The WHO recommends that disclosure should be conducted gradually, in line with the child’s age and their emotional maturity [45]. However, it is difficult for primary caregivers to implement this recommendation without a great deal of support from healthcare workers [3, 11, 45, 46]. Authors of a recent systematic review of disclosure of HIV status to children in sub-Saharan Africa reported that primary caregivers needed support from healthcare workers to effectively disclose HIV status to children [11]. Within the context of a trusting relationship, it is essential for primary caregivers to understand why it is important to disclose and to develop the skills necessary to do this in a safe and effective manner.

Another important finding in our study was that relationships of unequal power, misunderstandings, and the perception that primary caregivers have little knowledge about HIV among participants prevented open communication. In Malawi power is exercised through a variety of hierarchies that include bureaucracy, tradition and educational attainment [47]. The power of healthcare workers and teachers is based on their high level of education and their status as professionals. They are respected and regarded as important people in the community [47]. The majority of primary healthcare users are women with low socioeconomic status and lower social status than men. This reinforces the imbalance of power and constrains their engagement with healthcare workers, as well as participation in healthcare programmes [47].

In addition to their low social status, women who care for children living with HIV, are often treated rudely by healthcare workers because they have no choice in relation to the clinics they attend and are powerless to complain. There are few private healthcare facilities in the rural areas where most families reside, and the great majority of women would not be able to afford private care in any case. As such, healthcare workers in public facilities are in high demand. They sometimes exercise their authority by providing or withdrawing services or resources from the people they serve [47]. Inappropriate or rude actions usually go unpunished [47]. The unequal power between healthcare workers and the people they serve is highlighted in a number of studies conducted in Malawi [48–51]. For example, the authors of two studies that assessed women’s perception of antenatal care found that health care workers treated women who came for antenatal care as though they were children, they were shouted at and ordered not to complain otherwise they would be sent back home [48, 50]. In another study, family members who were giving basic care to very sick relatives reported that healthcare workers were disrespectful toward them and that they were often chased away from the wards where their relatives were patients [49]. In a related study, patients reported that they were sometimes slapped and swore at by healthcare workers [50]. In a final example, in a recent study about healthcare utilisation in Malawi, the authors found that the poor behaviour of healthcare workers was one of the reasons why some people decided to stop using public services altogether [51]. It is, therefore, no surprise we found that open communication between healthcare workers and primary caregivers was uncommon. This issue is not specific to Malawi, it is prevalent in all sub-Saharan countries [52–54] and also not unusual in resource-rich settings [55].

While it would be easy to blame healthcare workers for this lack of open communication and occasionally inappropriate behaviour, it is important to acknowledge that the healthcare workers, themselves, are neither, the true cause of, or the ultimate solution to, the problem. The underlying cause is a chronic lack of financial, physical and human capital resources in the healthcare system [56]. The majority of people in Malawi are extremely poor, and they cannot afford private healthcare. The services that are available are free and largely financed by the Government. However, due to the global economic downturn, the Government currently provides even less funding for healthcare services than in the past [56]. For example, in 2014 the Government of Malawi provided US$11 per person for basic health services instead of the US$86 that is recommended for Malawi [57]. In addition to a lack of physical resources, such as hospitals and equipment, insufficient funding has resulted in inadequate investment in human resources. For example, the ratio of professional healthcare workers to the population is 0.2 per 10,000 and 3.4 per 10,000 for doctors and nurses respectively [58]. The nurse to population ratio is only a third of what WHO recommends [58]. Inadequate funding has resulted in exceptionally high workloads and very low salaries for healthcare workers in Malawi [58, 59]. Because of high workloads, healthcare workers have reported feeling exhausted and failing to discharge their duties professionally [60].

It has been suggested that ensuring there is equal power and trusting relationships between primary caregivers and other groups of participants is an important step in providing a safe environment for HIV disclosure [61]. The building of trust helps to promote engagement between community members and healthcare workers [61–63]. Patients, or members of the public, need to trust healthcare workers before they engage with them in any important health interventions [63]. It is a necessary step towards building a good relationship that can lead to a free and open discussion [61]. In addition, trust in the healthcare system helps to improve patients’ health outcomes and satisfaction with care [64]. It is, therefore, important for healthcare workers to create a conducive environment where primary caregivers can trust them to discuss their concerns regarding the disclosure of HIV status [45]. This sounds ideal, and yet the reality is that it is very difficult for healthcare workers in Malawi to make the time, and have the composure required to communicate effectively with primary caregivers about HIV disclosure with their children.

Participants in this study reported the importance of shared responsibility among the participants in order to meet the needs of children living with HIV. It was suggested that the four groups of participants make a disclosure plan together and develop strategies to protect the child from the negative consequences of disclosure. For participants to work together, there is a need for them to have a common language and a mutual understanding which can be made possible through more open channels of communication [65, 66]. Shared responsibility ensures accountability among stakeholders since each member is aware of the boundaries of his or her responsibility and that of others in the team [65]. It also ensures the continuity of care since a channel of communication is established to ensure the flow of information regarding the care that the child is receiving and the next plan of care [65].

According to Durch and colleagues [66], in the context of health promotion, the first step to shared responsibility is for the participants to acknowledge that all the groups of participants share the responsibility to improve the health of a particular population. The second step involves assigning specific tasks to each group of participants. Each group participants could be held accountable for the assigned task. Successful performance could be rewarded, and failures be subjected to review by the participatory groups. The assignment of tasks helps to develop a “round table” approach rather than a top-bottom approach [66]. In addition, shared responsibility should be built on inclusion, trust, respect relationships, and equality among all members [67]. The team members need to put in place a clear guideline on how the partnership will work and identify which channel of communication team members will follow [65]. The members will also need to choose active and effective team leaders to coordinate the activities, as often partnerships with passive leaders do not last long [68].

In our study, teachers and community leaders reported that they could not adequately support children living with HIV because primary caregivers did not inform them about their HIV status. Similar findings were reported in a study from Zimbabwe, where teachers reported that they were not told about the HIV status of the pupils who were living with HIV, which made it difficult for them to identify them and provide the necessary support [23]. The failure of primary caregivers to inform their child’s teacher of their HIV status is not confined to sub-Saharan Africa. The authors of a study recently conducted in the UK reported that the primary caregivers of 89% of children living with HIV did not inform schools about their children’s HIV status which made it difficult for teachers to support the children [69]. The most important part of ensuring a workable partnership to support children living with HIV is social disclosure. According to the WHO, social disclosure entails that the primary caregiver identifies and shares the child’s HIV status with other people in his/her social network who may assist in the disclosure process and provision of other related issues [1]. For this to be possible mutual trust and understanding among the team members is important [70]. Primary caregivers need assurance that shared information will be kept confidential and that their child will be protected from the negative consequences of disclosure. As they mature emotionally and cognitively, children themselves need to become involved in the process so that they are aware of the kind of support they can receive from each member of the team [1].

Participants suggested that one way of sharing responsibility was providing care to children through support groups. Adolescents reported that joining a support group was a key strategy that facilitated their acceptance of HIV. Moreover, the support group helped them to develop resilience to stigma and discrimination and adopt a healthy lifestyle. Similar findings are reported in studies from Zimbabwe, Botswana, and Thailand [12, 28, 71, 72]. Other benefits of support groups to children as reported by authors of recent studies include helping children to understand their illness [12]; regain their confidence [28]; and receive support related to education, skill building, stigma, healthy living, and love [72].

To the best of the authors’ knowledge, this is the first study to be conducted in Malawi and sub-Saharan Africa to assess the involvement of key stakeholders in the disclosure of HIV status to children. The study aimed to address gaps in knowledge about HIV disclosure to children that had been identified by WHO [1]. It is anticipated that the study findings will bring the issue to the attention of people throughout the world and make a significant contribution to the practice and the development of policies and materials related to paediatric HIV care not only in Malawi but also in other sub-Saharan African countries.

The study has a number of strengths. Firstly, participants represented all main stakeholder groups involved in the care of a child living with HIV. Second, they came from all regions in Malawi which are diverse in terms of culture and socioeconomic status. Third, data collection involved both interviews and focus group discussions which helped to provide rich data. Fourth, the large numbers of interviews and focus groups meant that data saturation was easily reached. Fifth, data were analysed using a well-accepted systematic approach. The study is not without limitations. First, due to the nature of the study, subjectivity during data analysis and interpretation cannot be ruled out. Nonetheless, the process of data analysis involved a group of experts with a wide range of expertise who reviewed and identified themes, and this helped to validate and improve the credibility of the study findings. Second, the findings may be prone to social desirability bias considering that we collected data from well-respected people in the community who are supposed to live an exemplary life and thus are likely to provide anticipated correct responses.

## Conclusion

The findings of this study have shown that there is agreement among stakeholders about the need to work together to disclose HIV status to children. The provision of ARV medication to children at no direct cost to their families has resulted in a marked reduction in new cases of HIV among children and substantially increased the life-expectancy of children with the disease. There is now great hope for many children to lead fulfilling lives and contribute significantly to the economic development of their country. While this success in the prevention and treatment of HIV is to be applauded, there are consequences for children living with HIV and their families that have not been fully recognised. One of the key issues is that many children are growing up with no knowledge that they have HIV [9, 73–75]. These children are at increased risk of a range of poor health and developmental outcomes [76, 77] and they pose a risk to others through the inadvertent spread of HIV. Therefore, in addition to ARV medication, these children and their families, as well as local health and education services, require a great deal of support with appropriate physical and human resources. Healthcare workers and teachers would benefit greatly from training in working together with families living with HIV and, specifically, training in the disclosure process. They also require resources, in the form of books and other educational materials, to help explain HIV and effective management to children and families. While this may seem to be a relatively straightforward goal, it is an enormous task to achieve in Malawi and other sub-Saharan African countries where healthcare budgets are already stretched beyond the limit, and there are numerous health and social problems.

## Abbreviations

AIDS: Acquired Immuno-Deficiency Syndrome
ART: Antiretroviral Therapy
ARVs: Antiretrovirals
DVD: Digital Video Disc
FPAM: Family Planning Association of Malawi
HIV: Human Immunodeficiency Virus
MOH: Ministry of Health
NAPHAM: National Association of People Living with HIV and AIDS in Malawi
NGO: Non-Governmental Organizations
UK: United Kingdom
UNAIDS: Joint United Nations Programme on HIV/AIDS
UNESCO: The United Nations Educational, Scientific and Cultural Organisation
UNICEF: United Nations International Children’s Emergency Fund
US: United States of America
WHO: World Health Organization
